# The Southern Dental Association and the Dental Colleges

**Published:** 1873-09

**Authors:** 


### ARTICLE II.

### *The Southern Dental Association and the Dental Colleges.*

Southern Dental Association, at the Richmond Meeting, 1872.

*Resolved*, That in furtherance of the object of this resolution the President of this Association be requested to appoint a committee of one from each of the Southern States, the President of the Association, *ex-officio*, being chairman, whose duties it shall be to correspond with the faculties of the Baltimore and New Orleans Dental Colleges in relation thereto, and ascertain from them upon what terms either or each of them will consent to a reorganization of their faculties, or an amalgamation of the two colleges, and in consideration of a compliance with either of these terms, the college so reorganized will be adopted and endowed by this Association; that the said committee be instructed to bring this enterprise prominently before the members of the profession in their respective States, and at the same time request of their brethren what amount they will subscribe towards this endowment, and report to the Association the whole matter at our next annual meeting in Baltimore."

ABINGDON, VA., February 10th, 1873.

*To the Dean of the Faculty of the Baltimore College of Dental Surgery:*

DEAR SIR:—By the terms of a resolution adopted by the Southern Dental Association at its last annual meeting, it is made my duty as *ex-officio* chairman of the Committee on Professional Education and Dental Colleges, to open a correspondence with the Faculties of the Baltimore and New Orleans Dental Schools, and submit the following enquiry and proposition for their consideration, and report the result to the next annual meeting to be held in the city of Baltimore, July, 1873, viz:

Will the Faculty of your School consent to a reorganization; and if judged expedient, and promotive of the interest of dental education, to a combination and consolidation of the two Southern Colleges, viz: The Baltimore and New Orleans, upon the condition and assurances that the School so formed from the elements of the two above named Institutions, and such other materials as may be available and desirous, shall be adopted, and as liberally as may be, endowed by the said Southern Dental Association, &c.?

In executing the duty confided to me, it is proper, and no less due the Body I represent, than the Faculties of the Schools I am commissioned to approach, that a clear and distinct explanation of the motives and reasons which prompted the action of the Association should be made; and to be full and explicit, to place the whole matter beyond the pale of rational misapprehension, we will first state that the Association did *not* mean by its action in the premises. It did not mean to reflect injuriously or disparagingly upon either the New Orleans or the Baltimore School It did not mean to apply any curt censure, or express any hostility or unfriendliness for either of the Schools, or the individuals composing their respective Faculties; it did not mean to discriminate against either, or in favor of one against the other, nor against both in favor any enterprise of the future. So much for the negative consideration.

But the Southern Dental Association did recognize the fact, and did mean to express the conviction, that a well organized and appointed, an efficiently officered, and liberally *endowed* and supported dental college, was an indispensable necessity in the South; and further, that under existing circumstances, in view of the depleted and exhausted condition of our section, it was impossible to sustain more than *one* such institution. The demands of the present, are for an institution with *all* the facilities and appliances, for the most thorough and complete instruction of dental students in all the departments of the profession; for a college upon a firm, a fixed and *established* basis, commanding and *compensating* the most distinguished talent in our department of science,—an institution independent of the dictation of patrons, and of the accidents and circumstances which often diminish the support of such school below the self-sustaining point.

That for thirty or more years, since the first legal and public recognition of dentistry as a science, the profession have been dependent upon the *unrequited* and *uncompensated* labors of the noble and self-sacraficing men who have generously and faithfully filled our *chairs*, and benevolently trained and instructed our students; that such service could not reasonably be relied upon, as indefinite and perpetual, and should not in *honor* continue a tax upon the time and labor of our benefactors, without our looking to some measure that would reward their zeal and fidelity to a profession which claims to be "liberal."

That the present *status* and condition of our educational relations might be earnestly given as follows: We in the South have two colleges, both honored and valued institutions, both challenging our admiration and enlisting our support and sympathies, each necessarily conflicting and competing with the other, both trammeled in their usefulness, restricted and crippled in their growth and influence; both languishing for support, and almost struggling for existence. That to correct this condition of our educational interest, a combination and consolidation of our schools, a concentration of the patronage, the influence and the *pecuniary resources* of the profession in the South, appeared the most feasible remedy,—and without intending or desiring to infringe or infract any individual or corporate right, privilege or franchise, without the remotest idea or feeling of discourtesy, or without the slightest purpose of officiously intermeddling with the individual or corporate concerns of either School, concluded to submit to your consideration and determination, the only plan which appeared available for the object contemplated.

Having placed the whole matter as we understand it, fully before you, will you have the kindness to submit the propositions of the Southern Dental Association with the accompanying explanations, to a meeting of your Faculty of instruction, or your Board of Visitors, as may be proper, and at your earliest convenience advise us as to the results, so that I may have ample time to make up my report for the ensuing meeting of the Association. With distinguished regard I have the honor to be

Your very obedient servant, &c.,

H. M. GRANT, M. D., D. D. S.

*President of Southern Dental Association.*

**(The same was sent to the Dean of the New Orleans College.)**

---

### ANSWER OF THE BALTIMORE COLLEGE OF DENTAL SURGERY.

BALTIMORE, *April* 23rd, 1873.

*Pres. of Southern Dental Association.*

DEAR SIR:—Your letter as Chairman of the Committee appointed by the Southern Dental Association at its last annual meeting held in Richmond, Va., relating to the two Southern Dental Colleges, was received and presented to the Faculty of the Baltimore College of Dental Surgery, at a recent Faculty meeting, when it was respectfully and carefully considered, and the Dean instructed to reply as follows:

Whereas, the members of the Faculty of the Baltimore College of Dental Surgery are laboring for the interests of the profession of Dentistry, and have a sincere desire to advance its *status* by every means in their power, and maintain and increase the usefulness of the institution they represent: therefore be it

*Resolved*, that a Chair of "Microscopical and Comparative Anatomy" be established, and a Professor be appointed to discharge the duties pertaining to such a chair, provided the Southern Dental Association will endow the same, and its members use their influence in advancing the interests of the Baltimore College ofDental Surgery, so long as they are satisfied that the Faculty of this institution are striving by all the means in their power to maintain and advance the *status* of Dentistry.

*And be it further resolved,* That recognizing in Prof. S. P. Cutler, D. D. S., M. D., of Holly Springs, Miss., a gentleman in every respect qualified by scientific research and attainment to perform the duties relating to a professorship of Microscopical and Comparative Anatomy, he be appointed as the professor of this branch of the Sciences in the Baltimore College of Dental Surgery.

Respectfully, &c.,

F. J. S. GORGAS, M. D., D. D. S.

*Dean of the Faculty.*

---

### ANSWER OF THE NEW ORLEANS DENTAL COLLEGE

NEW ORLEANS *May* 7th 1873.

*Pres. Southern Dental Association.*

DEAR SIR:—In conformity with the preamble and resolutions recently passed by the Faculty of the New Orleans Dental College, I herewith transmit to you a copy of the same.

Whereas, at the last meeting of the Southern Dental Association, held at Richmond Va., the idea was then advanced that too great a number of Dental Colleges existed, which prevented them from receiving that encouragement and support which such institutions deserve, thereby inducing a competitive rivalry, which naturally tends to lower the standard of ability in their graduates by too often conferring degrees upon incompetent candidates: and

Whereas, it was also contended that their limited support did not permit them to employ the best talent for the occupancy of their professional chairs, and to remedy these supposed evils in the South, it was proposed to abrogate one or the other of the two Dental Colleges in the South, [the Baltimore and the New Orleans Dental Colleges,] or to merge them into one, which is to be located at some convenient point, and then to endow the institution thus organized ; and now to carry out this intention, a committee was appointed to confer with the Faculties of these respective institutions to ascertain on what terms such an arrangement might be made ; therefore be it

*Resolved*, That this Faculty regards the premises assumed at that meeting as being wholly false and untenable, and the proposition made to abrogate the New Orleans Dental College an impertinent and unwarrantable request.

In the first place no rivalry whatever exists, situated so widely apart as they are, between the Baltimore and the New Orleans Dental Colleges. As an evidence of this fact many students attend lectures in this city who would have gone to Philadelphia ; and there are, on the other hand, a larger portion of others who enter the Dental College, who, were it not for the convenience thus afforded, would not have been Dental Students. So far from diverting students from Baltimore, not more than three or four, since the establishment of the New Orleans Dental College, now over six years, have attended its lectures who would have gone to the former ; on the contrary, several attendants at this College have gone to Philadelphia to complete their studies, notwithstanding they had been urged to go to Baltimore for that purpose.

Instead of competition acting disadvantageously to the success of collegiate institutions, the very opposite effect obtains ; for it is well known that students increase in numbers wherever foci for collegiate instruction are established. In proof of which, witness the extensive, the quadrupled patronage which either of the two Dental Colleges in Philadelphia now annually receives, to what it was when only one existed. The same thing occurred in New Orleans in respect to medical colleges. Neither can this faculty believe that healthy competition can in any way impair the efficiency in dental colleges, or cause a deterioration in the quality of their instructors.

*Resolved*, That New Orleans being most eligibly situated for a dental college, commanding by natural as well as artificial communications the whole South-West, and having the States of Mississippi, Arkansas, Louisiana, Texas, and the largest part of Alabama, to which will soon be added California, as direct tributaries for furnishing pupils to her Dental College, its Faculty can not entertain any proposition towards its discontinuance, especially after having weathered all financial difficulties, and become self-sustaining—in fact established upon a firm, permanent basis. Nor are they willing to sacrifice by so doing, the large sums of money that were expended in its organization. The Faculty of this College, performing as they do the duties of Professors without compensation, fling back with contempt the insinuation that unworthy persons are ever graduated by this College merely for the sake of the accompanying fees.

*Resolved*, That the only arrangement that can be made between the Baltimore and the New Orleans Dental Colleges is a fusion of the former Institution with the latter; the junction thus effected to be located in New Orleans, and its corps of Professors to be selected equally from their present organizations.

*Resolved*, That from the already established character and usefulness of the Baltimore College, and from the advantageous position of the one in New Orleans, in being able to attract pupils from farther South and West, whose course would naturally tend Northward, the profession in the education of the future dentists of the South, can ill dispense with either of the Institutions named; but it is to the interest of the Southern Dental Association to extend protection and encouragement equally to both.

*Resolved*, That the Dean of this Faculty be requested to transmit a copy of this preamble and these resolutions to Drs. H. M. Grant, of Abingdon, Va., Chairman of the Committee on Dental Colleges; F. Y. Clark, Savannah, Ga., representing the State of Louisiana on said committee; and to Prof. F. J. S. Gorgas, Dean of the Baltimore Dental College, with a request that he have them published in the *American Journal of Dental Science*.

Very truly yours,

JAS. S. KNAPP D. D. S.

*Dean of the New Orleans Dental College.*

For the action of the Association and the discussion which followed the presentation of the report of the committee, the reader is referred to the proceedings of the late meeting, which are published in the present number of this Journal.

---

### ARTICLE III.

### *Physiology of the Dental Structures.*

